# Phosphoinositide 3-Kinase Alpha-Dependent Regulation of Branching Morphogenesis in Murine Embryonic Lung: Evidence for a Role in Determining Morphogenic Properties of FGF7

**DOI:** 10.1371/journal.pone.0113555

**Published:** 2014-12-02

**Authors:** Edward Carter, Gabriela Miron-Buchacra, Silvia Goldoni, Henry Danahay, John Westwick, Malcolm L. Watson, David Tosh, Stephen G. Ward

**Affiliations:** 1 Department of Pharmacy and Pharmacology, University of Bath, Bath, United Kingdom; 2 Centre for Regenerative Medicine, Department of Biology and Biochemistry, University of Bath, Bath, United Kingdom; 3 Novartis Institute of Biomedical Research, Horsham, United Kingdom; Cincinnati Children's Hospital Medical Center, United States of America

## Abstract

Branching morphogenesis is a critical step in the development of many epithelial organs. The phosphoinositide-3-kinase (PI3K) pathway has been identified as a central component of this process but the precise role has not been fully established. Herein we sought to determine the role of PI3K in murine lung branching using a series of pharmacological inhibitors directed at this pathway. The pan-class I PI3K inhibitor ZSTK474 greatly enhanced the branching potential of whole murine lung explants as measured by an increase in the number of terminal branches compared with controls over 48 hours. This enhancement of branching was also observed following inhibition of the downstream signalling components of PI3K, Akt and mTOR. Isoform selective inhibitors of PI3K identified that the alpha isoform of PI3K is a key driver in branching morphogenesis. To determine if the effect of PI3K inhibition on branching was specific to the lung epithelium or secondary to an effect on the mesenchyme we assessed the impact of PI3K inhibition in cultures of mesenchyme-free lung epithelium. Isolated lung epithelium cultured with FGF7 formed large cyst-like structures, whereas co-culture with FGF7 and ZSTK474 induced the formation of defined branches with an intact lumen. Together these data suggest a novel role for PI3K in the branching program of the murine embryonic lung contradictory to that reported in other branching organs. Our observations also point towards PI3K acting as a morphogenic switch for FGF7 signalling.

## Introduction

Branching morphogenesis is a key developmental process for the formation of many epithelial organs (lung, pancreas, mammary and salivary glands). During this process, epithelial tubes undergo successive rounds of coordinated branching. This culminates in an elaborate network of branched epithelial tubes that resembles the architecture of the adult organ. In the lung, this process occurs during the pseudoglandular stage of its development (embryonic day (E) 9–16 in mice and 5–17 weeks of human pregnancy) [1], [2].

The branching program of the lung requires highly organised cross talk between the branching epithelium and the surrounding mesenchymal tissue. To facilitate this process, the epithelium and mesenchyme secrete signalling molecules. Of particular importance for the branching epithelium are the fibroblast growth factor (FGF) family of growth factors. FGFs comprise a family of 22 factors that bind four types of tyrosine kinase receptors (FGFR1–4) [3]. Coordinated regulation of over 48 splice variants of the FGF receptors and interactions with tissue-dependent heparan sulphate molecules confers increased ligand and tissue specificity for different FGFs. Integral to the branching process is FGF10 that is produced by specialised mesenchymal cells ahead of a developing branch [4], [5]. FGF10 signals through epithelial FGFR2b to induce the budding and elongation of the branching epithelium [4], [6]. The importance of this pathway is highlighted by observations from mice deficient in either FGF10 [7] or FGFR2b [8], [9]. These mice form initial trachea and primary lung buds but lack any distal lung branching. FGF7 is also found during the branching program of the lung where it appears to contribute to the growth and differentiation of the lung epithelium [6], [10]. Mice lacking FGF7 do not exhibit any lung abnormalities suggesting a redundancy for FGF7 in the branching program [11].

As the FGFR2b receptor is ubiquitously expressed in the lung epithelium, signalling of FGF10 has to be restricted to the sites of branching. This is achieved by two means. Firstly, cells producing FGF10 only appear in the lung mesenchyme ahead of a developing branch [4]. However, this may be dispensable as ubiquitous expression of FGF10 across the lung mesenchyme fails to perturb epithelial branching [12]. Secondly, FGF10 signalling is restricted to sites of branching through binding to locally expressed heparan sulphate [13]. Indeed, addition of heparan sulphate to cultures of isolated epithelium perturbs the natural gradient thereby allowing for FGF10 to act ubiquitously across the lung epithelium. This results in FGF10 inducing uniform growth of the epithelium comparable to that of the related growth factor FGF7 [13], [14]. Similarly, altering the sequence of FGF10 to render the heparan binding domain inert creates a factor whose morphogenic responses are equal to that of FGF7 [15].

Phosphoinositide 3-kinase (PI3K) is a pleiotropic signalling molecule that influences cell proliferation, motility and survival. Four distinct subfamilies exist based on their substrate specificities and structures. Of these the class I family, comprised of the alpha, beta, delta and gamma isoforms, are the most extensively studied. The alpha and beta isoforms are ubiquitously expressed in mammals whereas the delta and gamma isoforms are mainly expressed in leukocytes [16], [17]. Class I PI3K enzymes phosphorylate the D3 position on the inositol ring of phosphatidylinositol 4,5-bisphosphate [PI(4,5)P_2_] to generate PI(3,4,5)P_3_ which controls a crucial signaling cascade implicated in a plethora of cellular responses [18]. A number of pan- and isoform selective PI3K inhibitors have been developed and are under current investigation for the treatment of cancers where the PI3K/Akt/mTOR pathway is central to the transformed phenotype of most cancer cells [19].

PI3K can be activated downstream of tyrosine kinase receptors such as FGF receptors where it can signal through the classical PI3K/Akt/mTOR pathway [20]. This signalling pathway has been found to be dysregulated in many inflammatory diseases [19], cancer [21] and fibrosis [22] where it contributes to the disease process. In development, PI3K-dependent signalling has been implicated in regulating epithelial branching in a number of organs [23]. For example, in cultures of murine salivary gland explants, inhibition of PI3K signalling with the pan class I inhibitors LY294002 and wortmannin greatly reduced epithelial branching. This inhibition of branching could be partially restored with the exogenous application of PI(3,4,5)P_3_, the main product of PI3K [24]. Moreover, PI3K and its downstream effectors Akt and Rac1 localise to the sites of, and were required for, branch initiation and elongation during the formation of mammary epithelial branches in response to hepatocyte growth factor [25]. Deletion of the alpha isoform of PI3K in murine mammary glands impaired the branching response whereas mice deficient in the beta isoform exhibited enhanced branching [26]. This would imply a divergent role for PI3K isoforms in this system. In contrast, the role of PI3K in airway epithelial branching is less clear. Murine lung explants treated with the pan-PI3K isoform inhibitor LY294002, exhibited reduced branching, implying a requirement for PI3K-dependent signalling as observed in other systems [27]. However, there is contrasting evidence to suggest a negative role for PI3K. In isolated lung epithelium derived from mice deficient in the 3′ lipid phosphatase PTEN (and thus exhibiting enhanced PI3K signalling) branching in the presence of FGF10 compared to wild type epithelium was significantly impaired [28].

In this present study we sought to dissect the role of individual class I PI3K isoforms in the branching program of the murine embryonic lung. To achieve this, we utilised isoform-specific PI3K inhibitors in an *ex-vivo* model of murine embryonic lung branching. To complement this approach we also examined downstream effectors of PI3K. We also examined the effects of PI3K inhibition directly on the lung epithelium with regards to the morphogenic properties of FGF7. We found that inhibition of PI3K alpha (or its downstream signalling components) could potentiate the branching of murine embryonic lung explants. We also demonstrate that PI3K inhibition in isolated lung epithelium is sufficient to alter the morphogenic properties of FGF7.

## Materials and Methods

### Inhibitor compounds

The class I PI3K inhibitor ZSTK474, the PI3K delta inhibitor IC-87114, the mammalian target of rapamycin complex (mTORC) 1/2 inhibitor AZD8055 and the mTORC1 inhibitor rapamycin were purchased from Selleckchem (Suffolk, UK). The PI3K gamma inhibitor AS-605240 was sourced from Cambridge Bioscience (Cambridge, UK). Akt inhibitor VIII was acquired from Merck chemicals (Dramstadt, Germany). The PI3K beta inhibitor GSK2636771 was obtained from Axon Medchem (Groningen, The Netherlands). The PI3K alpha inhibitor BYL719 was provided by Novartis (Basel, Switzerland).

### Murine embryonic whole lung explant culture

All animal experiments were performed within UK Home Office regulations and with the approval of the University of Bath ethics committee. E12.5 lungs were dissected from CD1 mouse embryos as previously described [29]. Isolated lung explants were placed on a Nuclepore track etched membrane (8 µm pore size; Fisher Scientific, Paisley, UK) suspended on DMEM: F12 culture medium (Fisher Scientific) supplemented with 0.5% FBS and placed in a 37°C 95% air/5% CO_2_ incubator. Images were collected using a Canon EOS 1100D digital SLR camera attached to an Olympus CKX41 inverted light microscope. Branching was quantified by the percentage increase in the number of terminal branches over 24 and 48 hours relative to the number at isolation using the equation: (Terminal branches_24/48 hours_- Terminal branches_0 hours_) ÷ Terminal branches_0 hours_ ×100.

### Isolated lung epithelium culture

E12.5 embryonic lungs were prepared as described above and the mesenchyme was stripped from the epithelial branches. Isolated mesenchyme-free epithelium was embedded in growth factor reduced Matrigel (BD Bioscience, Oxford, UK) mixed 1∶1 with DMEM: F12 cell culture medium supplemented with 0.5% FBS. Following Matrigel polymerisation at 37°C isolates were covered in culture medium and 100 ng/ml FGF7 (Miltenyi Biotech, Surrey, UK).

### Immunoflourescence

Lung explant cultures were fixed in MEMFA (3.8% formaldehyde, 0.15 M MOPS, 2 mM EGTA, 1 mM MgSO_4_, pH 7.4) and permeabilised with 1% Triton-X 100 (Sigma-Aldrich, Poole, UK). Isolated epithelial cultures were first removed from culture using Matrigel recovery solution (BD Bioscience) before being fixed with 4% paraformaldehyde and permeabilised with 0.05% saponin.

Samples were then blocked with 2% BSA in PBS and incubated with either mouse anti-E-Cadherin (Cat No: 610182; BD Bioscience), rabbit anti-SOX9 (Cat No: AB5535; Millipore, Watford, UK), mouse anti-Ki67 (Cat No: 610968; BD Bioscience), rabbit anti-phospho-histone H3 (PH 3; Cat No: 05-806; Millipore), rabbit anti-TTF1 (Nkx2.1; Cat No: WRAB-1231; Seven Hills, Cincinnati, USA) or rabbit anti-pro-surfactant protein C (SPC; Cat No: WRAB-9337; Seven Hills). Samples were then incubated with either FITC conjugated horse anti-mouse (Vector labs, Birmingham, UK) or Alexa Flour-568 donkey anti-Rabbit (Invitrogen, Paisley, UK) secondary antibodies prior to mounting. Where indicated, Nuclei and F-actin were visualised by incubating the samples with DAPI and rhodamine-conjugated phalloidin (Sigma-Aldrich) respectively prior to mounting.

Fluorescent images were acquired using a Zeiss LSM 510 confocal microscope.

### Western blot analysis

Murine lung explants were lysed in buffer containing 50 mM Tris-HCl, 150 mM NaCl, 1% Nonidet P40, 1 mM sodium vanadate, 1 mM sodium molybdate, 10 mM sodium fluoride, 40 µg/ml PMSF, 0.7 µg/ml pepstatin A, 10 µg/ml aprotinin, 10 µg/ml leupeptin and 10 µg/ml soybean trypsin inhibitor. Samples were then diluted in sample buffer containing 5% β-mercaptoethanol, boiled, and separated by electrophoresis on a 10% SDS–PAGE gel. Proteins were subsequently transferred to a nitrocellulose membrane and probed with either rabbit anti-phospho-Akt (Ser473) (Cat No: 4060S; Cell Signaling), rabbit anti phospho-S6 ribosomal protein (Ser235/236)(Cat No: 2211S; Cell Signaling), rabbit anti-Akt (Cat No: 9272; Cell Signaling) or rabbit anti-ERK (Cat No: SC-93; Santa Cruz, Middlesex, UK). Membranes were then incubated in an HRP-conjugated goat anti-rabbit secondary antibody (DAKO) before bands were visualised using an EZ-ECL chemiluminescence detection kit (Geneflow, Staffordshire, UK) with an ImageQuant developer (GE healthcare, Buckinghamshire, UK).

### Statistical analysis

Change in branching over 24 and 48 hours between groups was compared by two-way ANOVA followed by Dunnet's *post hoc* test.

The percentage of Ki67 positive cells was calculated using ImageJ software. Data are expressed as percentage of Ki67 positive cells relative to the total number of cells in one field of view. Significance between groups was determined by Student's t-test.

## Results

### PI3K/Akt signalling negatively regulates branching morphogenesis in the lung

**Figure 1:**
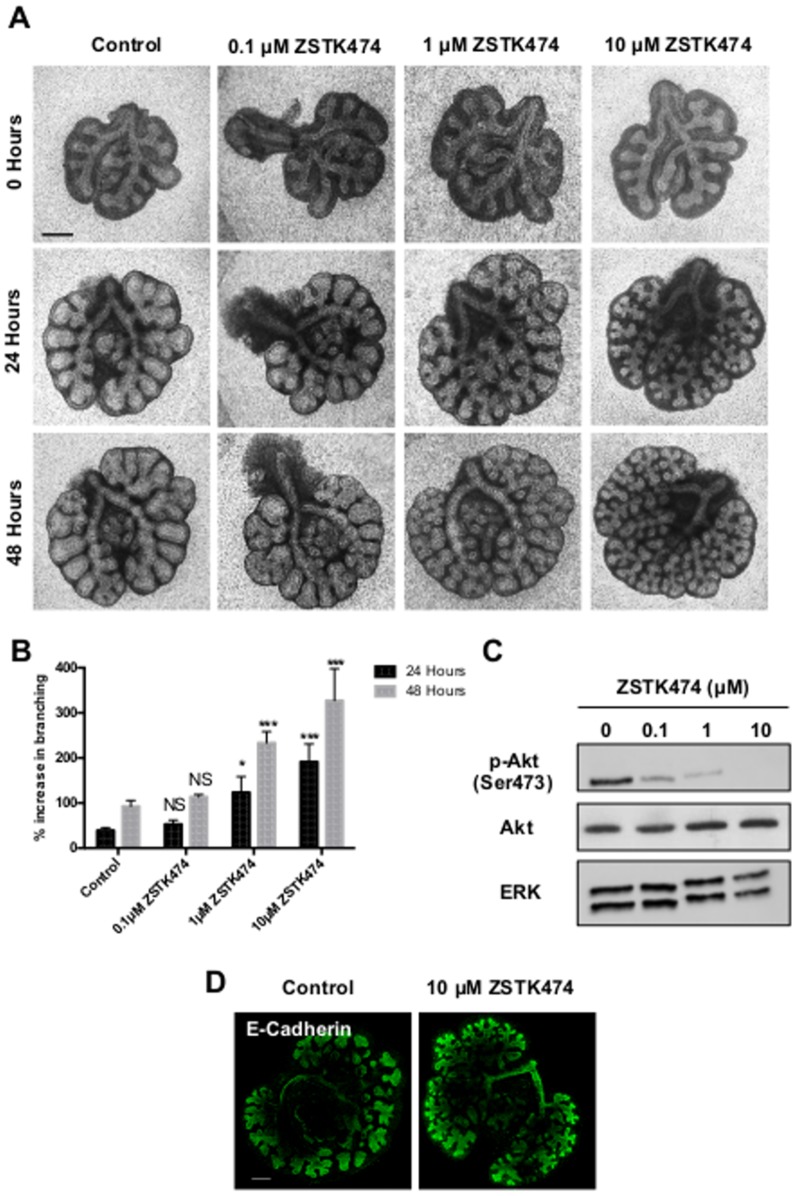
Inhibition of PI3K promotes epithelial branching in murine lung explants. **A.** Light microscope images of E12.5 murine lung explants cultured with either 0.1% DMSO (Control) or 0.1, 1 or 10 µM ZSTK474 over 48 hours. Images representative of at least 12 explants per condition are shown. Scale bar  = 0.5 mm **B.** Percentage increase in epithelial branching over 24 and 48 hours relative to the number of branches at initial isolation. Bars show mean ± s.e.m from n = 20 **C.** Western blot analysis for levels of phosphorylated Akt (p-Akt), total Akt and ERK in explants following 48 hours of culture. Total Akt and phosphorylated Akt bands are visualised from different gels containing an equal amount of the same protein lysate. Protein bands representative of 3 separate experiments. **D.** Confocal image of E-Cadherin expression in lung explants cultured with or without 10 µM ZSTK474 for 48 hours. Scale bar  = 200 µm. *** P<0.001 * P<0.05 * NS  =  Not Significant, compared with control.

To explore the role of PI3K signalling in branching morphogenesis, we used a panel of pharmacological tools with pan-isoform or isoform selectivity as well as inhibitors of various elements of the downstream signalling cascade. We first applied inhibitors of this pathway to whole explant cultures of murine embryonic lungs. Over 48 hours the pan class I inhibitor of PI3K, ZSTK474 [30], significantly increased branching in a concentration dependent manner compared to DMSO controls (Figure 1A, B). This enhancement of branching correlated with a concentration-dependent decrease in phosphorylated Akt (Figure 1C), an indirect measure of PI3K activity [31]. Immunofluorescence staining of the epithelial marker E-Cadherin also revealed that PI3K inhibition by ZSTK474 induced the formation of significantly more, albeit smaller, branches than controls (Figure 1D).

**Figure 2:**
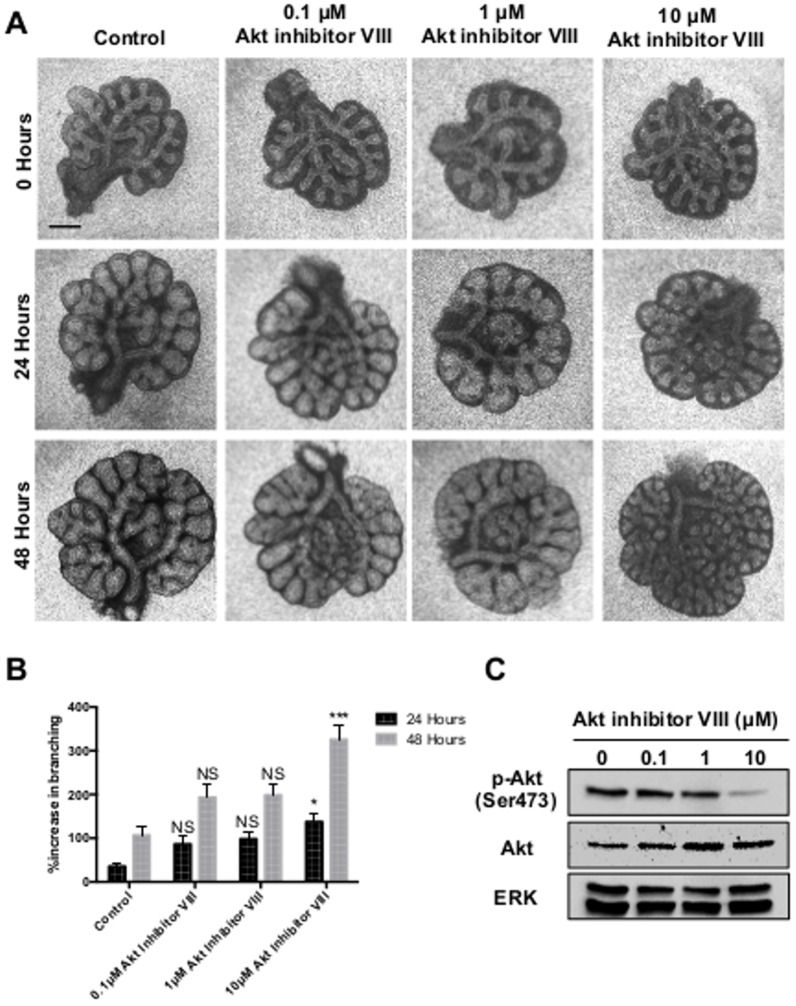
Inhibition of Akt promotes epithelial branching. **A.** Light microscope images of E12.5 murine lung explants cultured with 0.1% DMSO (Control) or 0.1, 1 or 10 µM Akt inhibitor VIII over 48 hours. Images representative of at least 12 explants for each condition are shown. **B.** Percentage increase in epithelial branching over 24 and 48 hours relative to the number of branches at initial isolation. Bars show mean ± s.e.m from n = 12 **C.** Western blot analysis for levels of phosphorylated Akt (p-Akt), total Akt and ERK in explants following 48 hours of culture. Protein bands representative of 3 separate experiments. Scale bar  = 0.5 mm. * P<0.05, *** P<0.001, compared with control.

**Figure 3:**
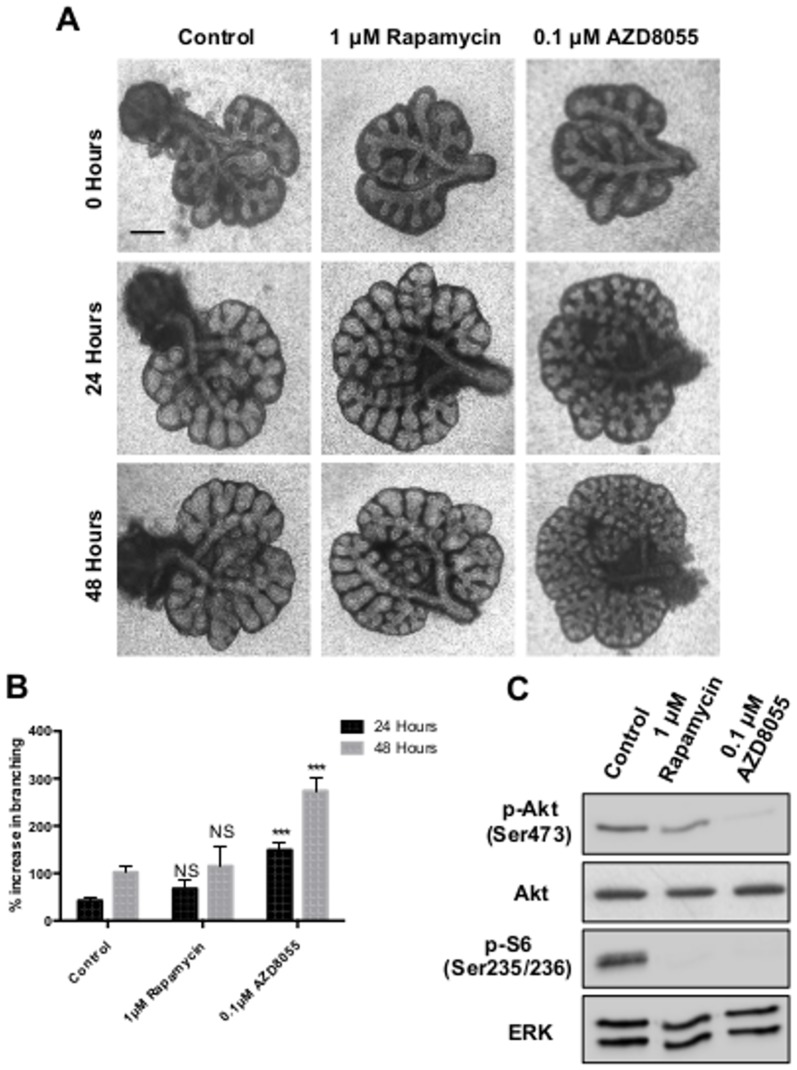
Inhibition of mTORC2 but not mTORC1 promotes epithelial branching. **A.** Light microscope images of E12.5 murine lung explants cultured in either 0.1% DMSO (Control), 1 µM rapamycin or 0.1 µM AZD8055. Images representative of at least 12 explants per condition are shown. **B.** Percentage increase in epithelial branching over 24 and 48 hours relative to the number of branches at initial isolation. Bars show mean ± s.e.m from n = 12. **C.** Western blot analysis for levels of phosphorylated S6 ribosomal protein (p-S6), phosphorylated Akt (p-Akt), total Akt and ERK in explants cultured for 48 hours. Total Akt and phosphorylated Akt bands are visualised from different gels containing an equal amount of the same protein lysate. Protein bands representative of 3 separate experiments. Scale bar  = 0.5 mm. *** P<0.001, NS  =  Not Significant, compared with control.

Consistent with an involvement of the PI3K pathway, we also observed enhanced branching when whole lung explants were cultured with an inhibitor of Akt, a primary signalling effector of PI3K. As with ZSTK474, Akt inhibitor VIII produced a concentration dependent increase in branching (Figure 2A, B). This increase in branching was accompanied by a reduction in phosphorylated Akt at the Ser473 position (Figure 2C). Phosphorylated Akt at Ser473 can be used as an indirect method of measuring Akt activation due to this residue being phosphorylated by mTORC2, which functions downstream of Akt [32]. Increased branching was also seen with the mTORC1/2 inhibitor AZD8055, but not with the mTORC1 inhibitor rapamycin (Figure 3A, B). Following activation of mTORC1 by Akt, mTORC1 phosphorylates S6 ribosomal protein; S6 ribosomal protein can therefore be used as an indicator of mTORC1 activity. Western blot analysis revealed a reduction in phosphorylated levels of S6 ribosomal protein following culture with either AZD8055 or rapamycin. However, only AZD8055 induced a loss in levels of phosphorylated Akt (Figure 3C). This enhancement of branching was also observed with the less-specific inhibitor of PI3K LY294002 at 10 µM (Figure S1). Conversely, a reduction in branching was noted at higher concentrations of the LY294002 compound (≥20 µM), similar to that seen in the literature [27].

### The alpha isoform of PI3K mediates branching in the murine lung

**Figure 4:**
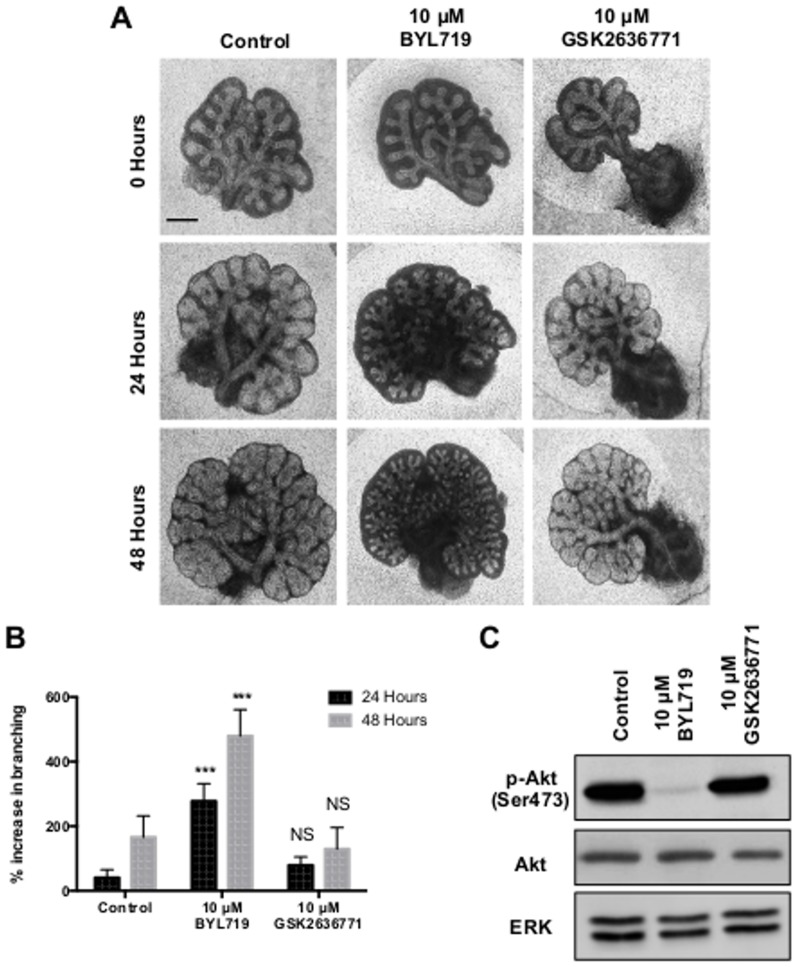
The alpha isoform of PI3K regulates lung branching morphogenesis. **A.** Light microscope images of E12.5 murine lung explants cultured with 0.1% DMSO (Control), 10 µM BYL719 or 10 µM GSK2636771. Images representative of at least 12 explants per condition are shown. **B.** Percentage increase in epithelial branching over 24 and 48 hours relative to the number of branches at initial isolation. Bars show mean ± s.e.m from n = 12. **C.** Western blot analysis for levels of phosphorylated Akt (p-Akt), total Akt and ERK in explants following 48 hours of culture. Total Akt and phosphorylated Akt bands are visualised from different gels containing an equal amount of the same protein lysate. Protein bands representative of 3 separate experiments. Scale bar  = 0.5 mm. *** P<0.001, NS  =  Not Significant, compared with control.

Given that the alpha and beta isoforms of PI3K have a divergent role in the branching program of the mammary gland [26], we sought to establish the roles of individual PI3K isoforms in lung branching. Whole lung explant cultures grown with a specific inhibitor of the alpha isoform of PI3K, BYL719 [33], produced a significant enhancement of branching (Figure 4A, B). Consistent with this, BYL719 induced a reduction in levels of phosphorylated Akt (Figure 4C). No alteration in branching was seen when whole explants were cultured with an inhibitor of the beta isoform of PI3K, GSK2636771 [34], over 48 hours (Figure 4A, B). Equally, no reduction in phosphorylated Akt was observed from explants cultured with GSK2636771, suggesting a lack of involvement of the beta isoform in this system. To confirm these findings we used other commercially available inhibitors specific for either the alpha or beta isoform of PI3K with different selectivity profiles. As with BYL719 an enhancement of branching was observed with the alpha inhibitor A66 [35]. No change in branching was seen at concentrations less than 10 µM with the beta inhibitor TGX-221 (Figure S2). A small enhancement in branching over controls was noted at higher concentrations of TGX-221 (e.g. >10 µM). However, this occurred at concentrations where other isoforms of PI3K would likely be inhibited [36]. Branching was unaffected following culture of explants with either the PI3K delta inhibitor IC-87114 or the PI3K gamma inhibitor AS-605240 (data not shown).

### FGF7-treated lung epithelium develop branches with a defined lumen and organised epithelium following PI3K inhibition

To determine if the effect of PI3K inhibition on branching was specific to the lung epithelium or secondary to an effect on the mesenchyme we assessed the impact of PI3K inhibition in cultures of mesenchyme-free lung epithelium. To confirm that mesenchyme stripping was efficient we examined isolated branch tips stripped of mesenchyme for the presence of residual mesenchymal cells (Materials and Methods S1). Confocal imaging demonstrated that freshly isolated epithelium contained cells that did not express the epithelial marker E-Cadherin. Cells that expressed the mesenchymal marker desmin were also apparent (Figure S3). Despite these residual mesenchymal cells isolated epithelium failed to grow without supplementation suggesting that the remaining mesenchyme was insufficient to maintain the viability of the epithelium (Figure S3).

**Figure 5:**
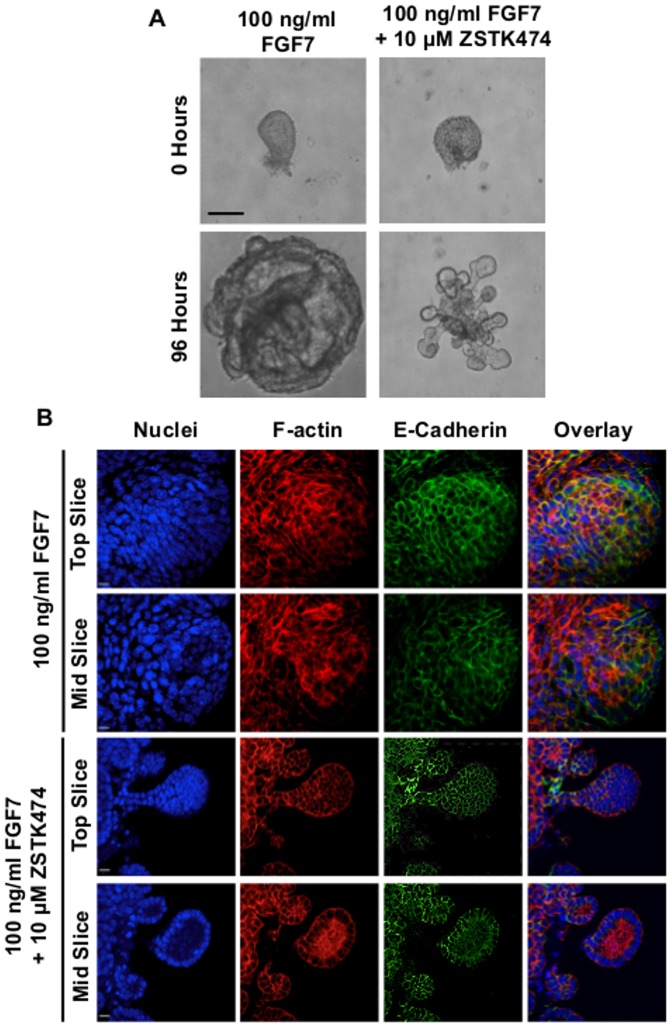
FGF7-treated lung epithelium develop branches with defined lumen and organised epithelium following PI3K inhibition. **A.** Light microscope images of isolated E12.5 murine lung epithelium cultured with either 100 ng/ml FGF7 alone (left panels) or in combination with 10 µM ZSTK474 (right panels) over 96 hours. 0.1% DMSO was used as a vehicle control along with FGF7. Images representative of at least 20 isolates are shown. Scale bar  = 200 µm. **B.** Z-stack confocal images of top and mid sections from isolated lung epithelium cultured with 100 ng/ml FGF7 alone or in combination with 10 µM ZSTK474 showing expression of the epithelial marker E-Cadherin and actin cytoskeleton. Images representative of three experiments. Scale bar  = 20 µm.

Isolated epithelium cultured in the presence of FGF7 formed large cystic structures after 96 hours (Figure 5A), consistent with the reported effects of this growth factor on cell proliferation [6]. Culture of epithelial isolates with FGF7 and ZSTK474 induced the formation of branch-like structures from the isolate (Figure 5A). To understand if these branched structures are fully formed branches or a simple scattering of cells we examined the morphology of the isolates by confocal microscopy. Z-stack analysis revealed that these structures contained intact lumen, as evidenced by the lack of nuclei in the centre of the branch, surrounded by cuboidal epithelial cells, features indicative of a fully formed branch (Figure 5B). Further, a patchwork of actin was noted at what appears to be a luminal surface (Figure 5B).

Similar to the effects observed with isolated lung epithelium co-cultured with FGF7 and ZSTK474, co-culture with FGF7 and the mTORC1/2 inhibitor AZD8055 elicited the appearance of branch structures from the initial isolate. Co-culture of FGF7 with the mTORC1 inhibitor rapamycin produced large cystic structures similar to that observed with FGF7 alone (Figure S4). This re-enforces the notion that mTORC1 alone is insufficient to promote branching in either the whole explant culture or in isolated lung epithelium.

### Inhibition of PI3K does not alter either the differentiation or the proliferation of distal lung epithelium in response to FGF7

**Figure 6:**
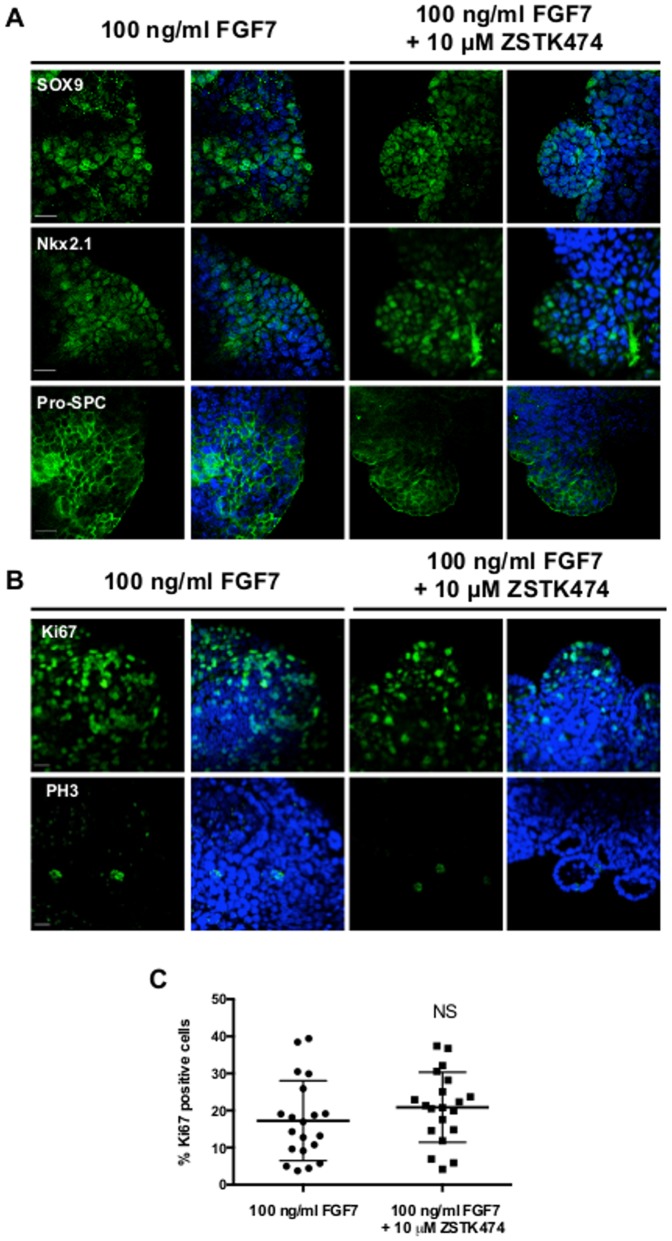
Inhibition of PI3K does not affect the differentiation of the lung epithelium nor does it affect their proliferation. **A.** Immunofluorescence images of the lung markers SOX9 (top panels), Nkx2.1 (middle panels) and pro-SPC (bottom panels) in isolated lung epithelium cultured for 96 hours with 100 ng/ml FGF7 with or without 10 µM ZSTK474. 0.1% DMSO was used a vehicle control along with FGF7. **B.** Immunofluorescence images of Ki67 (top panels) and phospho-histone H3 (PH3, lower panels) in isolated lung epithelium cultured over 96 hours with 100 ng/ml FGF7 alone or in combination with 10 µM ZSTK474. **C.** Percentage of Ki67 positive cells in isolated lung epithelium cultured with either FGF7 alone or in combination with ZSTK474. Data pooled from 20 images per condition. Images representative of at least 12 isolates per condition. Scale bar  = 20 µm. NS  =  Not Significant.

Next, we examined if the differentiation of the lung epithelium was been affected by inhibition of PI3K. Following culture with FGF7, the isolated lung epithelium ubiquitously expressed the marker of distal lung epithelium SOX9 [37]; a transcription factor required for distal lung morphogenesis, Nkx2.1 [38]; and a marker of differentiated lung epithelium, pro-SPC [6]. Expression of these markers was not appreciably changed in lung epithelium cultured with FGF7 and ZSTK474 (Figure 6A).

As the isolates cultured with ZSTK474 and FGF7 were smaller than those cultured with FGF7 alone we reasoned that proliferation may be reduced as PI3K inhibition has been reported to arrest cell growth [39]. To address this, we examined the expression of two markers of proliferation (Ki67 and PH 3) in isolated epithelium after 96 hours of culture with FGF7 alone or in combination with ZSTK474. Both isolates treated with FGF7 alone and with ZSTK474 showed uniform appearance of Ki67 after 96 hours of culture. Small, isolated, clusters of cell expressing PH 3 were also apparent in both culture conditions although these cells did not appear to be localised to specific regions (Figure 6B). Despite the isolates cultured with ZSTK474 and FGF7 being smaller in size than the isolates cultured with just FGF7, the proportion of Ki67-positive cells to the total cell number was not statistically different between the two groups (Figure 6C).

### Inhibition of PI3K reduces branching in embryonic pancreas

We wished to determine whether the observation with the PI3K inhibitors was specific to lung or whether it extended to other branching organs. To address this issue we isolated dorsal pancreatic buds from E11.5 mouse embryos and cultured these in the absence and presence of ZSTK474 (Materials and Methods S1). We used cytokeratin 7 (CK7) as a marker for the presence of pancreatic ductal branches. CK7 is an intermediate filament protein found in pancreatic ductal cells [40]. Under control conditions, CK7 stained ductular structures (Figure S5). However, treatment of dorsal buds with ZSTK474 suppressed the branching and the elongation of ductular structures based on the loss of the CK7 immunofluorescence (Figure S5).

## Discussion

In this study we investigated the role of PI3K signalling in lung branching morphogenesis. We show here, for the first time, that PI3K plays a negative role in airway branching morphogenesis. This has been demonstrated by a robust increase in murine lung explant branching upon pharmacological inhibition of either PI3K or its downstream effectors Akt and mTOR. Notably we observed a potentiation of branching with an inhibitor of both the mTORC1 and mTORC2 complexes of mTOR but not with an inhibitor of mTORC1 alone. Once activated mTORC2 can feedback and potentiate Akt activity [32] whereas mTORC1 can negatively regulate mTORC2 [41]. Thus inhibition of mTORC1 should increase Akt signalling by removing this inhibitory effect on mTORC2. In contrast, inhibition of both mTORC1 and mTORC2 would remove the negative feedback of mTORC1 but would also remove the positive effect of mTORC2. As a consequence the overall net effect would be decreased activation of Akt signalling and thus an increase in branching.

The use of isoform-specific inhibitors of PI3K suggests that the alpha isoform of PI3K negatively regulates lung branching. An increase in branching above that seen with the pan PI3K inhibitor was observed with the alpha specific PI3K inhibitors A66 and BYL719 but not with the beta specific inhibitors GSK2636771 or TGX-221. The enhancement seen with the alpha inhibitors over that observed with the pan inhibitor potentially reflects the increased potency of BYL719 and A66 for the alpha isoform compared to ZSTK474 at the concentrations used. An enhancement following inhibition of the alpha isoform, and no effect with inhibition of the beta, is in contrast to mammary glands where mice deficient in PI3K alpha exhibited decreased mammary branching [26]. Deletion of the beta isoform in the same study potentiated branching, pointing to divergent roles for the isoforms of PI3K. The distinct roles of PI3K alpha and beta may be explained by the fact that the beta isoform is less active and could compete with the more active alpha isoform for lipid substrate. Deletion of the beta isoform would therefore potentiate PI3K signalling through increased alpha isoform activity. We have also shown in the present study that inhibition of PI3K in mouse embryonic pancreas inhibits branching. Our work therefore represents a unique role for PI3K in the branching process of the lung, opposite to that seen in other branching organs. This has implications for the use of regenerative therapeutic targets that are directed at PI3K, as a promising effect in one organ may produce a deleterious effect in another.

The observation that inhibition of PI3K potentiates branching in the murine embryonic lung is somewhat surprising given that previous work has suggested a requirement for PI3K signalling. Wang et al. observed a reduction in branching in murine lung explants cultured with the pan class I PI3K inhibitor LY294002 [27]. In our study we observed a similar inhibitory effect with the LY294002 compound at concentrations used by Wang *et al.* (>20 µM). However, this was preceded by an efficient increase in branching at 10 µM. It therefore seems likely that at higher concentrations the actions of LY294002 on the lung explants are a consequence of toxicity and/or inhibition of branching through one of the many off-target effects of the compound [42].

**Figure 7:**
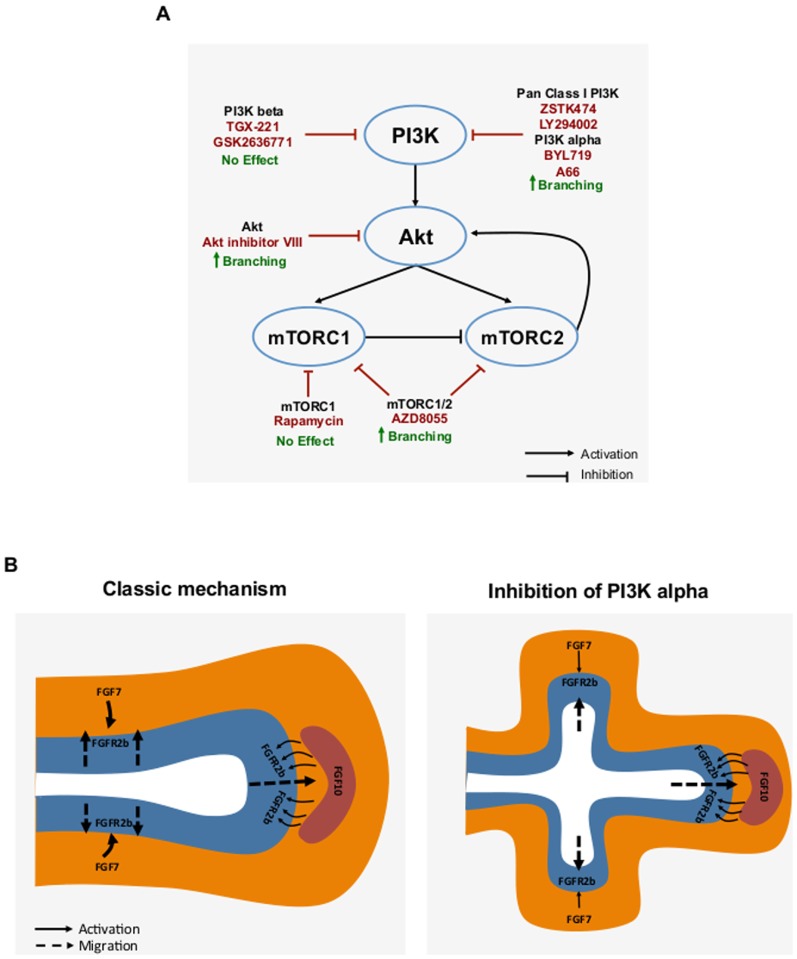
PI3K inhibition promotes branching morphogenesis through manipulation of FGF7 signalling. **A.** Sites of intervention for PI3K signalling and apparent effects on branching morphogenesis. Inhibitory compounds are presented in red text with apparent biological effect in green. Pharmacological inhibition of either PI3K alpha, Akt or dual inhibition of mTORC1/2 potentiates epithelial branching. Inhibition of either PI3K beta or mTORC1 has no observable effects on branching. **B.** FGF10 and FGF7 signalling during lung branching morphogenesis. Lung epithelium is displayed in blue surrounded by orange mesenchyme. Red represents FGF10 producing cells. Classically, FGF10 promotes branching whereas FGF7 induces the enlargement of the epithelium (left panel). In a setting where PI3K is inhibited (right panel) FGF7 signalling through FGFR2b is switched to an FGF10 signalling event. This has the outcome of inducing the formation of branches that would not otherwise develop.

Using a range of pharmacological agents, targeting PI3K and its downstream signalling components, we have been able to better explore the role of PI3K signalling in airway branching (Figure 7A). Thus our findings more accurately represent the role for the PI3K pathway in lung branching morphogenesis. Although the reported IC50 values for these compounds are usually in the nanomolar range these values are obtained from cell free assays and do not necessarily reflect their potency in cells and tissues. Thus, the higher concentrations required in our study to elicit inhibitory effects likely reflects the influence of cell membrane permeability and compound stability on inhibitor efficiency when using *in-vitro* cell/tissue based assays.

A particularly striking observation of this study is that branches form from isolated lung epithelium cultured with FGF7 when PI3K signalling is inhibited. These structures contain a luminal space and organised epithelium confirming that they are not merely a scattering of cells but are fully formed branches. This is the first reported observation of FGF7 promoting a branched response in isolated lung epithelium. As the isolates cultured with FGF7 and the inhibitor of PI3K ZSTK474 were smaller than those cultured with FGF7 alone we examined if proliferation was being affected. In the presence of FGF7 and ZSTK474 lung epithelial isolates continued to express the proliferation markers Ki67 and PH3. The proportion of cells expressing Ki67 was not significantly different between isolates cultured in the presence of FGF7 and ZSTK474 and those cultured with FGF7 alone suggesting that proliferation was not being altered. Interestingly the expression of PH3 was restricted to small populations of cells scattered across the epithelial isolate. This could suggest the presence of distal stem cells [43].

A requirement for PI3K in the signalling output of FGF10 and FGF7 through FGFR2b has recently been reported from Hela cells overexpressing this receptor. Stimulation of FGFR2b by FGF10 resulted in the p85 subunit of PI3K being recruited to the receptor separate from the classical Grb2/Gab2 interactions. Following internalisation, this interaction with PI3K led to the receptor being recycled to the cell membrane. In contrast, FGF7/FGFR2b signalling failed to recruit PI3K to this site and, upon internalisation, the receptor was degraded. Removal of this PI3K binding site from FGFR2b resulted in the degradation of the receptor following FGF10 simulation, producing a signalling outcome similar to FGF7 [44]. PI3K, acting as a switch for FGFR2b trafficking, therefore appears to be a critical determinant for the signalling output of a ligand. It is worth noting that inhibition of PI3K with LY294002 was shown to promote the recycling of FGFR2b following stimulation by FGF7 [44]. This work therefore lends a possible explanation as to how inhibition of PI3K is sufficient to promote branching from isolated lung epithelium cultured with FGF7. When PI3K signalling is inhibited, the FGFR2b receptor would be recycled, not degraded, following stimulation by FGF7. The net effect of this would then be an FGF10-like signalling event, i.e. a branching response.

FGF10 and FGF7 both act to regulate branching in the lung and salivary glands. In the lung, FGF10 promotes branch initiation and elongation [4] whereas FGF7 promotes the growth of the epithelium [6]. In the salivary gland FGF10 serves to promote bud elongation and FGF7 promotes branch initiation [15]. Differences in FGF actions on the branching epithelium may therefore account for the reported differences in the function of PI3K in the salivary glands and the lungs. In the salivary gland inhibition of PI3K would shift the action of FGF7 from a budding response to an elongation response (FGF10). This reduction in budding may have the net effect of reducing branching which is what is seen when PI3K is inhibited in salivary gland explant cultures [24].

We propose that, in a setting where PI3K signalling is inhibited in the developing airways, FGF7 produced in the mesenchyme acting through FGFR2b is modulated to produce a signalling outcome like FGF10. This would have the net effect of inducing the formation of branches that would not otherwise develop (Figure 7B). Local reduction in PI3K activity in the branching epithelium may therefore add a novel mode of regulation during branching morphogenesis.

Developmental pathways that orchestrate the growth of an organ can show a resurgence in disease settings. These developmental pathways control the remodelling of an organ to a degree where function becomes impaired. An example of this is transforming growth factor β, which is critical for lung growth. Too much or too little of this factor greatly perturbs branching. Signalling of this factor is also overactive in fibrosis where it is seen as one of the predominant pathogenic signals [45]. Insight into how signalling pathways (that act to remodel an organ during disease) function in the development of an organ will aid in the understanding of how the remodelling occurs and will lead to novel therapeutic strategies for regenerative medicine.

## Supporting Information

Figure S1 **LY294002 induces contrasting effects on lung branching.** **A.** Light microscope images of E12.5 murine lung explants cultured with 0.1% DMSO (Control) or 10, 20 or 30 µM LY294002 over 24 and 48 hours. Images representative of at least 9 explants per condition are shown. Scale bar  = 0.5 mm **B.** Percentage increase in epithelial branching over 24 and 48 hours relative to the number of branches at initial isolation. Bars show mean ± s.e.m from n = 9 *** P<0.001, * P<0.05, NS  =  Not Significant, compared with control.
(TIFF)

Figure S2 **Inhibition of PI3K alpha but not beta enhance branching in murine lung explant cultures.** Percentage increase in branching of E12.5 murine lung explants cultured with 0.1% DMSO (Control) or 0.1, 1 or 10 µM of either the PI3K alpha inhibitors BYL719 (**A**) and A66 (**B**) or the beta inhibitors GSK2636771 (**C**) and TGX-221 (**D**) over 24 and 48 hours. Bars show mean ± s.e.m from n = 12. ***P<0.001 *P<0.05, compared with control.
(TIFF)

Figure S3 **Mesenchyme-stripped branch epithelium still show evidence of intact mesenchymal cells.** **A.** Light microscope images of an isolated E12.5 murine lung branch before (left panel) and after (right panel) mesenchyme removal. Scale bar  = 200 µm. **B.** Expression of E-Cadherin (top panels) and desmin (bottom panels) in isolated murine lung branches following removal of mesenchyme. Scale bar  = 20 µm. **C.** Light microscope images of isolated lung epithelium cultured over 96 hours without additional media supplementation. Images representative of 6 isolates. Scale bar  = 200 µm.
(TIFF)

Figure S4 **FGF7-treated lung epithelium develops branches following inhibition of mTORC1/2 but not mTORC1.** Light microscope images of isolated E12.5 murine lung epithelium cultured over 96 hours with 100 ng/ml FGF7 alone (left panels) or in combination with either 1 µM rapamycin (middle panels) or 0.1 µM AZD8055 (right panels). 0.1% DMSO was used as a vehicle control along with FGF7. Images representative of at least 12 isolates. Scale bar  = 200 µm.
(TIFF)

Figure S5 **PI3K inhibition reduces branching in embryonic pancreas.** **A.** Light microscope images of E11.5 embryonic murine pancreas cultured for 8 days with either 0.1% DMSO (Control) or 0.1, 1, or 10 µM ZSTK474. Black arrows point to epithelial branches following treatment with ZSTK474. **B.** Expression of cytokeratin 7 in embryonic pancreas following culture with ZSTK474. Scale bar  = 500 µm.
(TIFF)

Materials and Methods S1 **Murine embryonic pancreatic bud culture and pancreatic bud and mesenchyme-stripped embryonic lung immunofluorescence.**
(DOCX)
