# Supplementary figures and images for: Phosphoinositide 3-Kinase Alpha-Dependent Regulation of Branching Morphogenesis in Murine Embryonic Lung: Evidence for a Role in Determining Morphogenic Properties of FGF7
Source: PLoS One. 2014 Dec 2;9(12):e113555. doi: 10.1371/journal.pone.0113555 (PMC4251986; doi:10.1371/journal.pone.0113555)

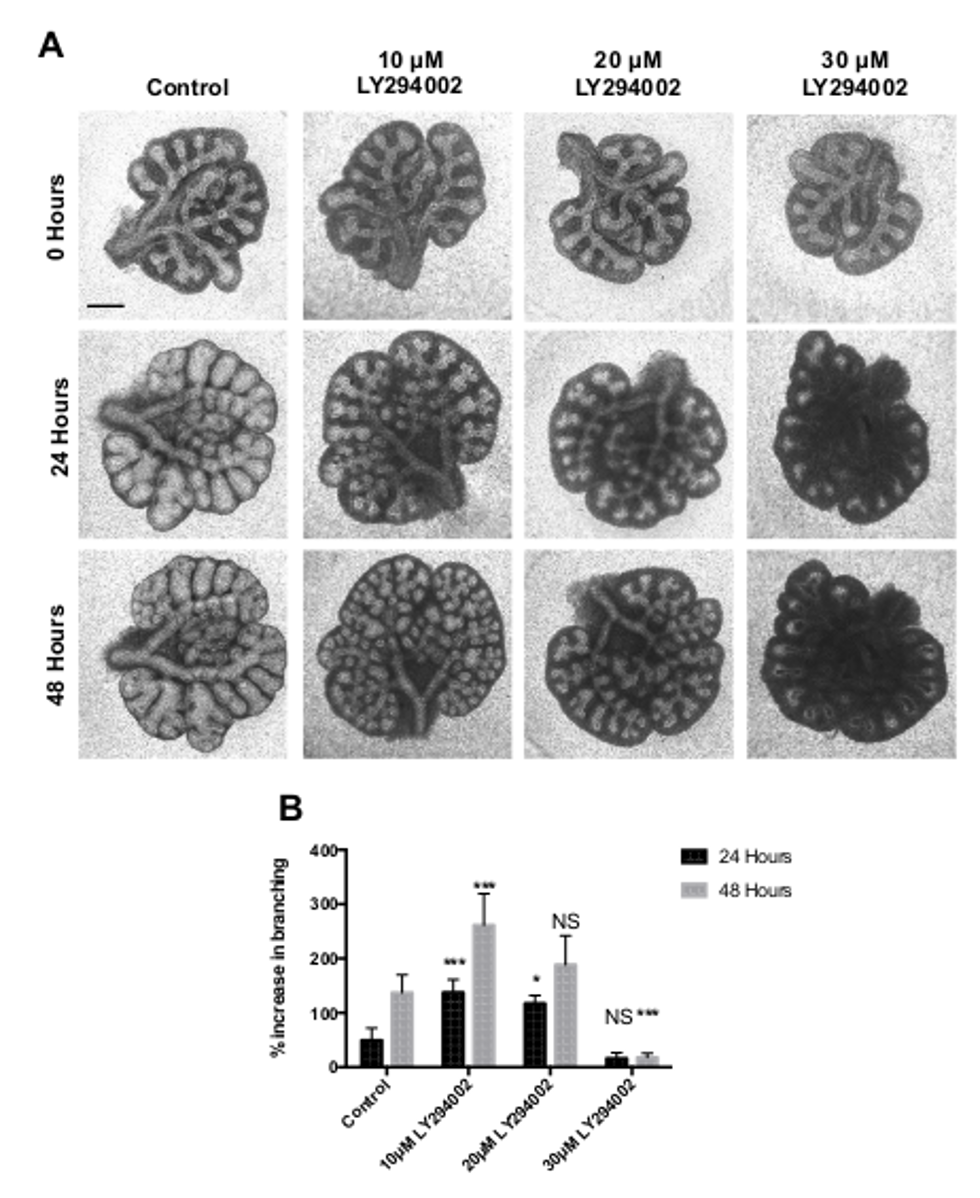

Supplement: Figure S1 — LY294002 induces contrasting effects on lung branching. A. Light microscope images of E12.5 murine lung explants cultured with 0.1% DMSO (Control) or 10, 20 or 30 µM LY294002 over 24 and 48 hours. Images representative of at least 9 explants per condition are shown. Scale bar = 0.5 mm B. Percentage increase in epithelial branching over 24 and 48 hours relative to the number of branches at initial isolation. Bars show mean ± s.e.m from n = 9 *** P<0.001, * P<0.05, NS = Not Significant, compared with control. (TIFF) [file pone.0113555.s001.tiff]

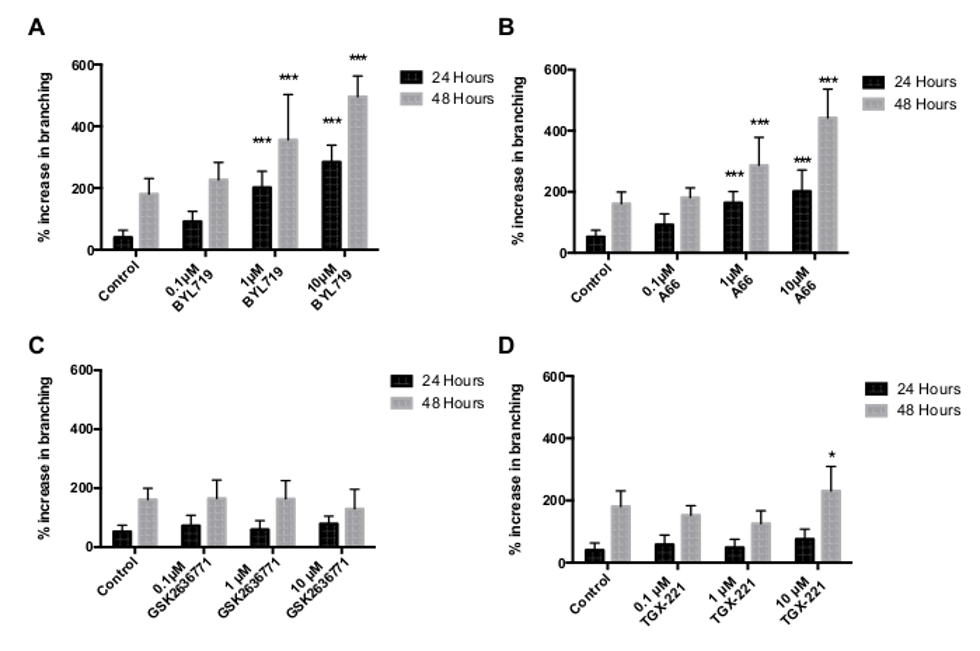

Supplement: Figure S2 — Inhibition of PI3K alpha but not beta enhance branching in murine lung explant cultures. Percentage increase in branching of E12.5 murine lung explants cultured with 0.1% DMSO (Control) or 0.1, 1 or 10 µM of either the PI3K alpha inhibitors BYL719 (A) and A66 (B) or the beta inhibitors GSK2636771 (C) and TGX-221 (D) over 24 and 48 hours. Bars show mean ± s.e.m from n = 12. ***P<0.001 *P<0.05, compared with control. (TIFF) [file pone.0113555.s002.tiff]

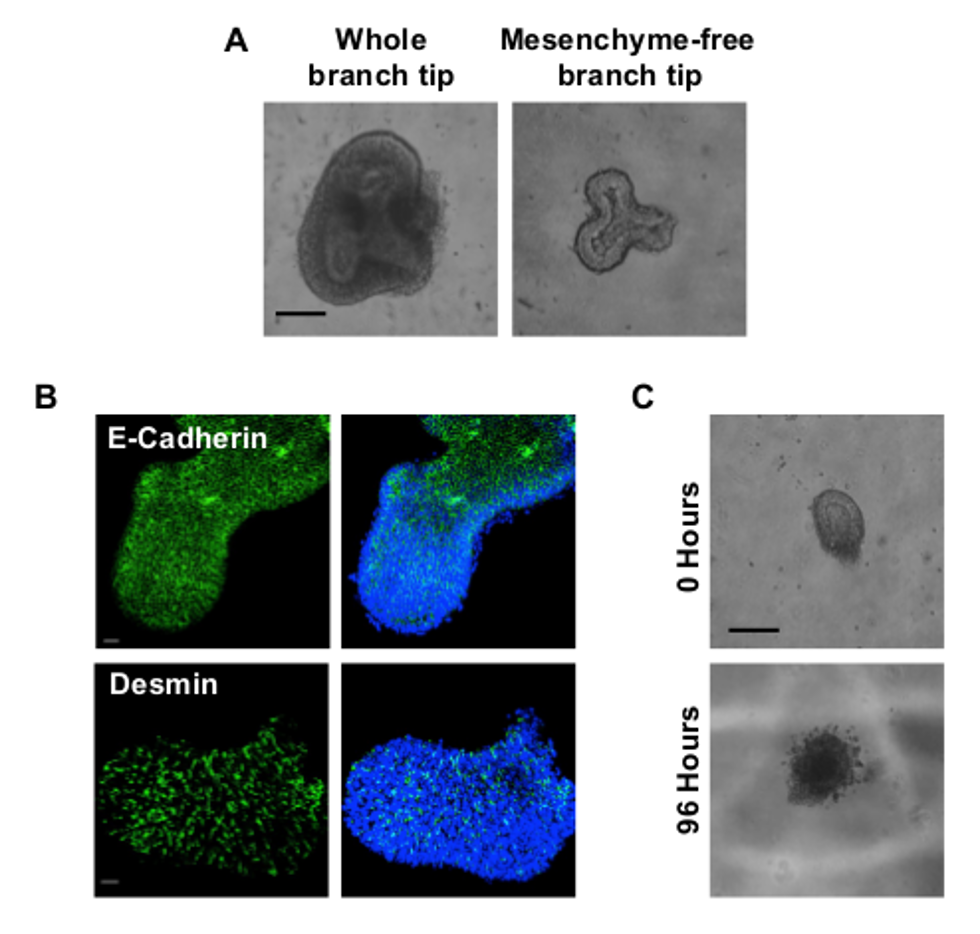

Supplement: Figure S3 — Mesenchyme-stripped branch epithelium still show evidence of intact mesenchymal cells. A. Light microscope images of an isolated E12.5 murine lung branch before (left panel) and after (right panel) mesenchyme removal. Scale bar = 200 µm. B. Expression of E-Cadherin (top panels) and desmin (bottom panels) in isolated murine lung branches following removal of mesenchyme. Scale bar = 20 µm. C. Light microscope images of isolated lung epithelium cultured over 96 hours without additional media supplementation. Images representative of 6 isolates. Scale bar = 200 µm. (TIFF) [file pone.0113555.s003.tiff]

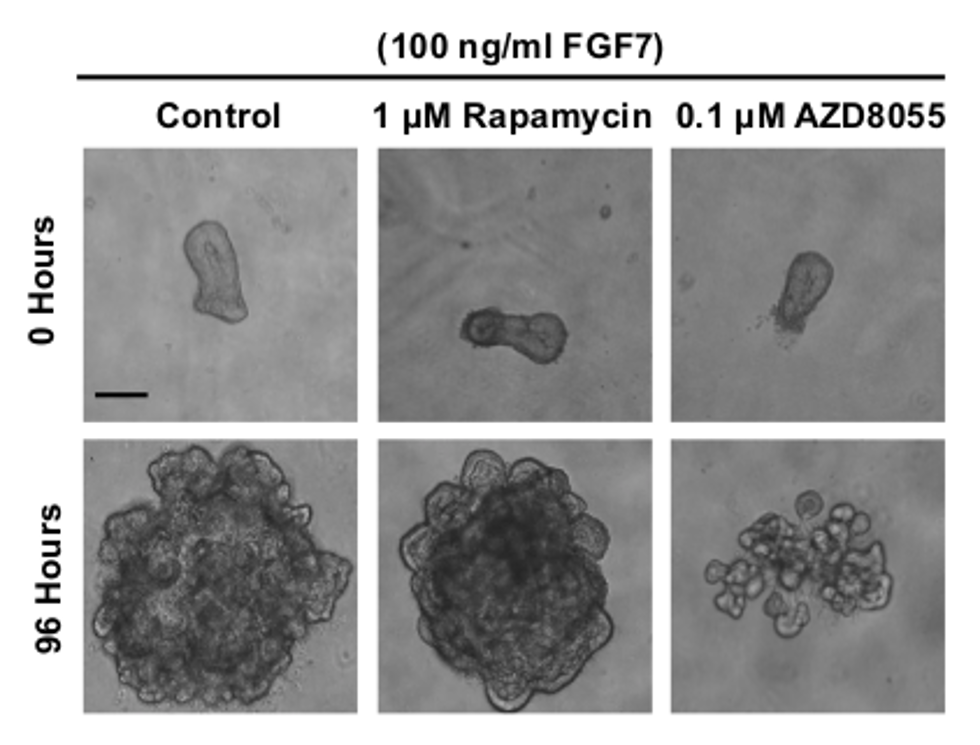

Supplement: Figure S4 — FGF7-treated lung epithelium develops branches following inhibition of mTORC1/2 but not mTORC1. Light microscope images of isolated E12.5 murine lung epithelium cultured over 96 hours with 100 ng/ml FGF7 alone (left panels) or in combination with either 1 µM rapamycin (middle panels) or 0.1 µM AZD8055 (right panels). 0.1% DMSO was used as a vehicle control along with FGF7. Images representative of at least 12 isolates. Scale bar = 200 µm. (TIFF) [file pone.0113555.s004.tiff]

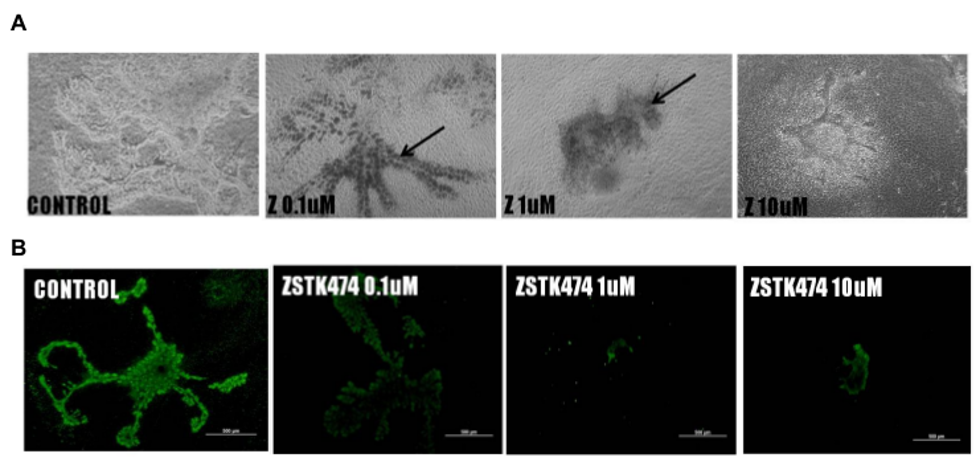

Supplement: Figure S5 — PI3K inhibition reduces branching in embryonic pancreas. A. Light microscope images of E11.5 embryonic murine pancreas cultured for 8 days with either 0.1% DMSO (Control) or 0.1, 1, or 10 µM ZSTK474. Black arrows point to epithelial branches following treatment with ZSTK474. B. Expression of cytokeratin 7 in embryonic pancreas following culture with ZSTK474. Scale bar = 500 µm. (TIFF) [file pone.0113555.s005.tiff]
