# Supplementary material for: Phosphoinositide 3-Kinase Alpha-Dependent Regulation of Branching Morphogenesis in Murine Embryonic Lung: Evidence for a Role in Determining Morphogenic Properties of FGF7
Source: PLoS One. 2014 Dec 2;9(12):e113555. doi: 10.1371/journal.pone.0113555 (PMC4251986; doi:10.1371/journal.pone.0113555)
Supplement: Materials and Methods S1 — Murine embryonic pancreatic bud culture and pancreatic bud and mesenchyme-stripped embryonic lung immunofluorescence. (DOCX) [file pone.0113555.s006.docx]

**MATERIALS AND METHODS S1**

**Compounds**

The pan class I PI3K inhibitor LY294002, the PI3K alpha inhibitor A66 and the PI3K beta inhibitor TGX-221 were sourced from Selleckchem (Suffolk, UK).

**Murine embryonic pancreatic bud culture**

Murine pancreatic buds were isolated and cultured as previously described (Thowfeequ et al., 2007). Briefly, E11.5 pancreatic buds were isolated from CD1 mice and cultured on fibronectin-coated coverslips (50 μg/ml) (Invitrogen, Paisley, UK). Pancreatic buds were maintained in BME cell culture medium (Sigma-Aldrich, Poole, UK) supplemented with 10% FBS, 1% L-glutamine and 100 U/ml penicillin and 100 μg/ml streptomycin. (Fisher Scientific, Paisley, UK) Pen/Strep and placed in a 37°C 95% air/5% CO_2_ incubator. Buds were treated without or with ZSTK474 (0.1, 1 or 10 μM).

**Pancreatic bud and mesenchyme-stripped lung immunofluorescence**

After 8 days of culture pancreatic buds were fixed with MEMFA and permeabilised with 1% Triton-X 100 (Sigma-Aldrich). Buds were then blocked in 2% blocking buffer (Roche, Hertfordshire, UK) before the addition of mouse anti-cytokeratin 7 (Cat No: ab9021; Abcam, Cambridge, UK) diluted 1/100 in 2% blocking buffer. Buds were incubated in primary antibody overnight before the addition of a FITC conjugated horse anti-mouse secondary antibody (Vector labs, Birmingham, UK) diluted 1/200 in 2% blocking buffer prior to mounting.

Mesenchyme-free lung epithelium were prepared as described in the materials and methods. These isolates were then fixed in ice-cold acetone: methanol (1:1). Isolates were incubated in either mouse anti-Desmin (Cat No: M076029-2; DAKO, Cambridge, UK) or mouse anti-E-Cadherin (Cat No: 610182; BD Bioscience, Oxford, UK) diluted 1/100 in PBS containing 2% BSA. Following incubation with primary antibody samples were incubated with FITC conjugated horse anti-mouse secondary antibody diluted 1/200 in PBS containing 2% BSA. Samples were finally incubated with DAPI prior to mounting.

**SUPPLEMENTARY REFERENCES**

**Thowfeequ, S., Ralphs, K. L., Yu, W.-Y., Slack, J. M. W. and Tosh, D.** (2007). Betacellulin inhibits amylase and glucagon production and promotes beta cell differentiation in mouse embryonic pancreas. *Diabetologia* **50**, 1688–1697.
